# Increase in Caesarean Deliveries after the Australian Private Health Insurance Incentive Policy Reforms

**DOI:** 10.1371/journal.pone.0041436

**Published:** 2012-07-23

**Authors:** Kristjana Einarsdóttir, Anna Kemp, Fatima A. Haggar, Rachael E. Moorin, Anthony S. Gunnell, David B. Preen, Fiona J. Stanley, C. D’Arcy J Holman

**Affiliations:** 1 Telethon Institute for Child Health Research, Centre for Child Health Research, University of Western Australia, Perth, Western Australia, Australia; 2 Centre for Health Services Research, School of Population Health, The University of Western Australia, Perth, Western Australia, Australia; 3 Centre for Population Health Research, Curtin Health Innovation Research Institute, Faculty of Health Sciences, Curtin University, Perth, Western Australia, Australia; 4 Health and Wellness Institute, Edith Cowan University, Joondalup, Western Australia, Australia; Aga Khan University, Pakistan

## Abstract

**Background:**

The Australian Private Health Insurance Incentive (PHII) policy reforms implemented in 1997–2000 increased PHI membership in Australia by 50%. Given the higher rate of obstetric interventions in privately insured patients, the reforms may have led to an increase in surgical deliveries and deliveries with longer hospital stays. We aimed to investigate the effect of the PHII policy introduction on birth characteristics in Western Australia (WA).

**Methods and Findings:**

All 230,276 birth admissions from January 1995 to March 2004 were identified from administrative birth and hospital data-systems held by the WA Department of Health. Average quarterly birth rates after the PHII introduction were estimated and compared with expected rates had the reforms not occurred. Rate and percentage differences (including 95% confidence intervals) were estimated separately for public and private patients, by mode of delivery, and by length of stay in hospital following birth. The PHII policy introduction was associated with a 20% (−21.4 to −19.3) decrease in public birth rates, a 51% (45.1 to 56.4) increase in private birth rates, a 5% (−5.3 to −5.1) and 8% (−8.9 to −7.9) decrease in unassisted and assisted vaginal deliveries respectively, a 5% (−5.3 to −5.1) increase in caesarean sections with labour and 10% (8.0 to 11.7) increase in caesarean sections without labour. Similarly, birth rates where the infant stayed 0–3 days in hospital following birth decreased by 20% (−21.5 to −18.5), but rates of births with >3 days in hospital increased by 15% (12.2 to 17.1).

**Conclusions:**

Following the PHII policy implementation in Australia, births in privately insured patients, caesarean deliveries and births with longer infant hospital stays increased. The reforms may not have been beneficial for quality obstetric care in Australia or the burden of Australian hospitals.

## Introduction

The Australian health care system has features of British and American systems; residents can access free treatment in public hospitals covered by national health insurance (public patients), or choose to be treated as private patients at either private or public hospitals at their own expense or at a subsidised cost through Private Health Insurance (PHI) [1], [2]. In an attempt to address the decline in PHI memberships among the Australian population the Australian government introduced strong tax-incentives in 1997–2000 to encourage the uptake of PHI. The incentives included the Private Health Insurance Incentive (PHII) scheme (1% tax-penalty for high income earners without PHI and a 30% tax rebate on PHI premiums) and the Lifetime Health Cover (LHC) (2% premium penalty pa for those who enter after the age of 30) [3], [4]. Following these policy reforms, the percentage of the population with PHI rose from 30% in 1999 to ∼45% in 2001 [5]. This increase has been attributed primarily to the introduction of the LHC as the 30% PHI rebate was reported to increase PHI coverage by only 1% from 1998 to 1999 [6], [7].

Considering that the PHII policy reforms were particularly targeted at younger people [8] and thus at women of childbearing age, it is likely there was an increase in the proportion of childbearing age women holding PHI. Since antenatal care in Australia is provided by private obstetricians for private patients and by rostered midwives, registrars and staff obstetricians for public patients, this may in turn have led to an increase in the number of women selecting to give birth as private patients due to the perceived benefits attributed to being a private patient (such as the ability to choose their own obstetrician). Given the higher rate of obstetric interventions such as caesarean deliveries, inductions, augmentations or instrumentally assisted deliveries observed in the private health sector [9], [10] the PHIIs may therefore have led to an increase in instrumentally and surgically performed deliveries and thus to an increase in length of hospital stay following birth.

Our objective was therefore to estimate average quarterly birth rates in Western Australia (WA) after the introduction of the PHIIs and compare it with rates that would have been expected had the policy not occurred. We calculated rate and percentage differences separately for public and private patients, by mode of delivery, and according to length of stay in hospital following birth.

## Methods

### Ethics Statement

The use of de-identified, administrative health data for this study without patient consent was approved by the Human Research Ethics Committee of the WA Department of Health. This study was performed in accordance with the Declaration of Helsinki.

### Data Sources

This study used routinely-collected, administrative health data from the WA Midwives Notification System (MNS) and the WA Hospital Morbidity Data Collection (HMDC), linked by the Data Linkage Branch at the WA Department of Health. The MNS data provided pregnancy and delivery details for all infants born in WA during 1995–2004 and the HMDC data provided hospital separation information for each birth that occurred in WA hospitals during 1995–2004.

### Study Population

Information from the MNS provided the basis for selection of the study cohort. The MNS is a statutory data collection which records information on all live or stillborn infants in WA of at least 20 weeks gestation or with a birth weight of at least 400 g. Multiple births (e.g. twins) were counted as one birth admission for this study, with the information on length of hospital stay for the first born infant being used. Also, births to both live-born and stillborn infants were included. Length of stay was categorized into 0–3 days and 4+ days following birth since most mothers and babies stay less than 4 days in hospital following an uncomplicated vaginal birth. The data did not have information on maternity services in WA and we were thus unable to assess the effect of the PHII reforms on access to services.

In addition to pregnancy and delivery details, the MNS provided information on the Index of Relative Socio-Economic (SE) Disadvantage (IRSD) based on maternal residence around the time of birth. The IRSD values are based on information on household income, educational attainment and occupation from the Australian census conducted every five years. The values were divided into quintiles for all analyses, with high scores reflecting low SE disadvantage in an area.

The information from the MNS on infant delivery details was linked with mothers’ hospital admission information from the HMDC to provide information on the funding source of the mother at the time of each hospital birth. Patient funding source was categorized to reflect two types of patients; those treated as public patients and those treated as private patients at time of delivery. Private patients were defined as those funded with PHI or who were self-funded, whereas public patients included those insured under the Australian national Medicare scheme.

### Statistical Analysis

We used logistic regression models to assess the difference in characteristics before and after the introduction of the PHII policy reforms and simple Chi square tests of independence for assessing the distribution of maternal age according to patient status, mode of delivery and length of hospital stay. The birth data was then analysed through interrupted time-series analyses using quarterly birth rates as main outcomes. Birth rates were estimated from the quarterly birth counts in our data (numerators) and the annual population figures for 12–50 year old females in WA (denominators) based on 5-yearly census data published by the Australian Bureau of Statistics (ABS) [11]. The ABS does not publish population figures for females by patient status or birth characteristics and we were thus not able to stratify the denominators by the variables under study. As such, we used the overall annual population figures for all rates.

Segmented regression analyses assuming the outcome rates followed Poisson distributions were used to measure the impact of the LHC [12]. The regression models included a term for the PHII policy implementation, which represented the first 18 months after the announcement of the LHC (Jan00– Jun01), the last policy of the PHIIs to be announced. This period was excluded from the time series analysis to account for health insurance funds’ waiting periods and the duration of pregnancy.

We used the segmented regression models to estimate the post-PHII average quarterly rates and compared them with the expected rates, calculated from the model as the projection of pre-PHII trends under the assumption that no intervention occurred [12]. Rate differences between the estimated and expected average quarterly rates and their respective percentage changes (including 95% confidence intervals) were calculated for overall birth rates as well as separately for birth rates in public and private patients, by mode of delivery and length of hospital stay. All analyses were performed using the statistical software SAS version 9.1 (SAS Institute Inc., Cary, NC, USA).

## Results

We included 230,276 birth admissions in this study that occurred from January 1995 to March 2004 in WA. Table 1 shows the characteristics of the 125,817 (55%) births that occurred before the introduction of the PHII policy reforms (January 1995-December 1999) and the 67,402 (29%) births that occurred after the reforms (July 2001-March 2004). Births to private patients, older mothers and mothers living in low SE disadvantage areas were slightly more common following the PHII policy reforms than before the reforms. All differences were statistically significant (p<0.0001).

**Table 1:** Characteristics of WA birth admissions before and after the introduction of the PHII policy reforms.

	Pre-PHII	Post-PHII	
	Jan95– Dec99	Jul01– Mar04	
	% (n = 125,817)	% (n = 67,402)	p-value^a^
Patient status			–
Public patient	69.56	64.3	
Private patient	30.4	35.66	<0.0001
Maternal age (years)			
12–24	24.1	21.7	
25–34	61.8	61.0	
35–50	14.2	17.3	<0.0001
SE disadvantage			
Low^b^	58.8	59.49	
High^c^	41.16	40.5	<0.0001

a Logistic regression analysis adjusted for all factors in the table.

b SE: Socio-economic. Quintiles 1–3.

c SE: Socio-economic. Quintiles 4–5.

In Table 2 we present the estimated average quarterly birth rates after the PHII introduction (July 2001-March 2004) and the average rates that would have been expected at the same time had the policy not been implemented. The results show that the PHII reforms were associated with only a small decrease (−1.3%) in birth rates overall compared with expected rates and although it was statistically significant this small decrease may have been due to the demographic trend of decreasing births in WA at the time. However, when the birth rates were estimated separately by patient status, the policy introduction was associated with a 20% decrease in births to public patients and a 50.7% increase in births to private patients. Also, a decrease in vaginal births, both unassisted and assisted (−5.2% and −8.4%, respectively), a 4.8% increase in caesarean sections with labour and 9.9% increase in caesarean sections without labour was observed after the PHIIs. Similarly, births where the infant stayed only 0–3 days in hospital following birth decreased by 20.0% following the policy implementation, whereas births where the infant stayed more than 3 days in hospital increased by 14.7% compared with expected estimates.

**Table 2:** Estimated average quarterly birth rates after the introduction of the PHII reforms (Jul01– Mar04) compared with rates that would have been expected at the same time had the policies not occurred.

	Estimated	Expected	Rate	Percentage
	quarterly	quarterly	difference	difference
	rates^a^	rates^a^	(95% CI)	(95% CI)
All	111.5	113.0	−1.5 (−2.0,−0.9)	−1.3 (−1.8,−0.8)
Patient status				
Public patient	70.6	88.6	−18.0 (−19.1,−17.0)	−20.3 (−21.4,−19.3)
Private patient	39.1	26.0	13.1 (12.1,14.1)	50.7 (45.1,56.4)
Mode of delivery				
Unassisted vaginal	64.6	68.2	−3.5 (−3.6,−3.5)	−5.2 (−5.3,−5.1)
Assisted vaginal	13.5	14.7	−1.2 (−1.3, −1.2)	−8.4 (−8.9,−7.9)
Caesarean with labour	11.9	11.4	0.5 (0.5,0.6)	4.8 (4.2,5.4)
Caesarean without labour	21.5	19.6	1.9 (1.5,2.3)	9.9 (8.0,11.7)
Length of stay in hospital				
0–3 days	45.8	57.2	−11.5 (−12.5,−10.4)	−20.0 (−21.5,−18.5)
4+ days	65.8	57.4	8.4 (7.1,9.6)	14.7 (12.2,17.1)

a per 10,000 population.

Given that private patients are generally older and that caesarean sections without labour and longer hospitals stays are more common on older mothers (Table 3), we additionally examined the association of the PHII policy reforms with maternal age at birth. Surprisingly, our results showed that estimated birth rates of mothers aged 12–24 years increased by 3.2% (2.6,3.8), whilst rates of mothers aged 25–34 and 35–50 decreased by 1.8% (−2.3,−1.4) and 8.7% (−8.7,−8.7), respectively, following the PHII introduction, compared with expected rates at the same time.

**Table 3:** Maternal age characteristics of WA birth admissions during January 1995-March 2004.

	Maternal age			
	12–24 years	25–34 years	35–50 years	
	% (n = 53,260)	% (n = 141,561)	% (n = 35,455)	p-value^a^
Patient status				–-
Public patient	91.8	62.8	53.2	
Private patient	8.2	37.2	46.8	<0.0001
Mode of delivery				
Unassisted vaginal	71.0	59.7	52.1	
Assisted vaginal	12.4	14.3	11.8	
Caesarean with labour	8.7	9.6	10.3	
Caesarean without labour	8.0	16.4	25.8	<0.0001
Length of stay in hospital				
0–3 days	55.3	38.7	31.5	
4+ days	44.7	61.3	68.5	<0.0001

a Chi square test of independence.

## Discussion

Our results show the association between the introduction of the Australian PHII policy reforms and changes in birth rates in WA during 1995–2004. Following the introduction of the PHIIs, birth rates in public patients decreased while birth rates in private patients increased, possibly as a result of a shift from public to private care. Our results also showed that vaginal deliveries decreased, caesarean deliveries increased and rates where the infant stayed longer than three days in hospital increased in the period following the PHII implementation. These associations did not appear to be due to increased birth rates in older mothers.

This study draws on the wealth of birth and hospital inpatient information routinely collected by the WA Department of Health. The MNS and HMDC are both statutory data collections and for the time period under study, we were able to study almost the complete birth information in WA since we received de-identified data from the WA Department of Health for 99.998% of all births recorded in the MNS for the entire state of WA. Despite the obvious strengths of using population based data such as this, we cannot be absolutely certain that our findings were caused by the PHII policy reforms. However, the increase in PHI uptake following the introduction of the government’s tax-incentives in 1997–2000 has been attributed primarily to the introduction of the LHC alone [6], [7]. This is evidenced by the fact that the 30% PHI rebate was found to increase PHI coverage by only 1% from 1998 to 1999 [6], [7]. As a result, and since no other major health insurance-related or obstetric policy reforms were introduced around this time, our results can most likely be attributed to the LHC introduction.

Our results indicated that following the PHII introduction, more women gave birth as private patients and more caesarean sections, particularly caesarean sections without labour, were performed, possibly as a result of this shift from public to private obstetric care. Our findings support previous research showing that privately insured women are more likely to have obstetric interventions than women treated in the public health system [9], [10], [13]. For instance, privately insured women in Australia have greater likelihood of receiving episiotomy [13], a higher probability of caesarean section or instrumentally assisted delivery [9], and a higher risk of forceps or vacuum delivery and of other obstetric interventions such as epidural anaesthesia, induction or augmentation than their public system counterparts [10]. Similar results are reported in the international literature, where midwife-led care is associated with fewer obstetric interventions than other models of care [14]. Although it is clear that adequate access to obstetric interventions such as emergency caesarean delivery can save the life of both the mother and infant [15], [16], high rates of operative delivery, particularly rates above 15%, may result in poorer maternal and infant outcomes for the current or subsequent births [17], [18], [19], [20], [21], [22], [23], [24], [25], [26], [27], [28], [29], [30]. With the rising caesarean section rates during the last few decades in the developed world, adverse outcomes following birth are gaining greater attention [31], [32], [33], [34], [35]. Betrán et al. analysed caesarean section rates both in developed and developing countries and found a strong inverse association between caesarean section rates and maternal, infant and neonatal mortality in countries with high mortality levels [18]. The authors stated that for developed countries with lower mortality levels the relationship becomes more ambiguous, but when caesarean section rates rise above 15%, risks of adverse health outcomes begin to outweigh the benefits [18]. Results on the relationship between caesarean section rates and mortality rates have not been previously published for WA, but analyses are underway in our research group to address this issue.

In Australia, caesarean section rates rose from 18% in 1991 [35] to 31% in 2008 [33], reaching the same prevalence as in the United States in 2006 [34]. It is likely that there are many reasons for this increase in caesareans section rates, including fear of litigation [36], maternal request [37], previous caesarean section [38], and as well, increase in the numbers of women with private health insurance. However, it appears clear from other studies that increases in maternal or foetal risk indicating the need for operative delivery are not a major factor [39], [40]. Due to the increased risk of injury and morbidity in the mother and infant following high rates of operative deliveries [17], [18], [19], [20], [21], [22], [23], [24], [25], [26], [27], [28], [29], [30], it seems clear that although the LHC policy may have been successful in achieving the government’s aim of relieving pressure on public hospitals [7], it may not have been beneficial for quality obstetric care in Australia.

Previous studies have found that length of hospital stay following childbirth is generally shorter in public hospitals than private hospitals in Australia [41], [42], as well as for other forms of midwifery-led care internationally [14]. Our results support these findings, as birth rates with longer hospital stays increased significantly following the PHII policy reforms in parallel with an increased use of private obstetric services. It is likely that our findings are due to the high probability of obstetrics interventions in the private system since early postnatal discharge has been found to be associated with lower levels of obstetric intervention [42]. However, our findings could also be explained by women’s preferences, as community surveys have indicated that new mothers have a preference for longer hospital stays following birth [43], [44], [45]. Previous studies have suggested that longer hospital stays do not appear to reduce adverse effects on infant feeding or maternal emotional health [46], [47], [48]. As such, it may appear that greater length of hospital stay is not clinically justified for healthy mothers and term infants, raising concerns regarding the likely influence on the economic burden on hospitals in Australia [49], [50].

In conclusion, this study assessed the impact of the PHII policy initiatives in 1997–2000 on birth rates in WA during 1995–2004. The results of our study reflect a shift away from public care (with greater midwifery input) towards obstetrician-led modes of care. The shift resulted in an increased rate of caesarean sections, particularly caesarean sections without labour, and in increased rate of births with longer hospital stays. The results indicate that the PHII implementation may not have been beneficial for obstetric care in Australia or the burden of Australian hospitals. Our findings are important for health care planning and policy, not only in Australia, but also in other countries where both public and private health insurance is available. The results illustrate the unforeseen and sometimes serious consequences that can occur following health care policy implementation in any country aiming to increase private health insurance membership. The lessons learnt in Australia can guide health care policy makers elsewhere in the world.
